# A219 BIOLOGIC USE DURING PREGNANCY IN WOMEN WITH INFLAMMATORY BOWEL DISEASE AND ASSOCIATED NEONATAL OUTCOMES: A RETROSPECTIVE CHART REVIEW

**DOI:** 10.1093/jcag/gwac036.219

**Published:** 2023-03-07

**Authors:** Y Jeyakumar, K O'Connor, V Huang

**Affiliations:** 1 Medicine, University of Toronto; 2 Gastroenterology, Mount Sinai Hospital, Toronto, Canada

## Abstract

**Background:**

Biologics are the mainstay of therapy for patients with advanced inflammatory bowel disease (IBD). Most biologics readily undergo placental transfer during the second trimester and remain detectable in infant serum for up to 12 months. Therefore, most clinical guidelines currently recommend avoiding live vaccines in the first 6 to 12 months for exposed infants. In Canada, the rotavirus vaccine is the only live vaccine administered before 6 months. Emerging evidence is suggesting that the vaccine may safely be given to infants with normal immune function, even if serum biologic level is detectable. Specialist assessment of infant immune function may help guide decision-making about rotavirus vaccine administration, but there remains a large gap in our knowledge of the impact of biologic exposure on the immune function of infants, necessitating further study.

**Purpose:**

In this single-centre retrospective chart review, we aim to study the clinical outcomes, immune function, and rotavirus vaccine recommendations for infants exposed to biologics in utero during their first year of life.

**Method:**

The study included mothers seen at the Pregnancy IBD Clinic at Mount Sinai Hospital in Toronto, ON, who were offered a referral to the Special Immunization Clinic (SIC) at The Hospital for Sick Children due to biologic exposure in utero. Data was collected on the recommendations made by paediatric specialists at SIC, based on complete blood count, lymphocyte phenotyping, and T-cell receptor excision analysis. Data was obtained on adverse neonatal outcomes in the first year of life, including prematurity, congenital malformations, and infections based on post-partum surveys completed by mothers and follow-up letters from SIC.

**Result(s):**

43 patients were referred to and seen by paediatric specialists at SIC between 2 to 12 months of age. 18 infants (42%) were exposed to Adalimumab in utero, 16 (37%) to Infliximab, 5 (12%) to Vedolizumab, and 4 (9%) to Ustekinumab. The rotavirus vaccine was recommended to 34 infants (81%) and not recommended to 3 (7%) for reasons including gastrointestinal illness, neutropenia, and low lymphocyte counts. Recommendation is pending for 6 infants (14%). Two infants (5%) had premature births. Four infants (9%) were admitted to the NICU for reasons including respiratory distress, and prematurity (9%). One infant (2%) had a congenital malformation, specifically bilateral sensorineural hearing loss. Six infants (14%) had upper respiratory tract infections, none of which required hospital admission or antibiotics.

**Conclusion(s):**

This study is currently in progress. Further data is required to assess whether biologic exposure in utero has a significant impact on neonatal immune function, especially beyond the first year of life, and whether there is a significant difference in vaccine recommendations and response based on the type of biologic received.

**Disclosure of Interest:**

None Declared

